# Genetic differentiation between sandfly populations of *Phlebotomus chinensis* and *Phlebotomus sichuanensis* (Diptera: Psychodidae) in China inferred by microsatellites

**DOI:** 10.1186/1756-3305-6-115

**Published:** 2013-04-22

**Authors:** Li Zhang, Yajun Ma, Jiannong Xu

**Affiliations:** 1 Department of Pathogen Biology, Second Military Medical University, Shanghai, China; 2 Department of Biology, New Mexico State University, Las Cruces, NM, USA

**Keywords:** *Phlebotomus chinensis*, *Phlebotomus sichuanensis*, Genetic differentiation, Microsatellites, China

## Abstract

**Background:**

*Phlebotomus chinensis* is a primary vector of visceral leishmaniasis; it occurs in various biotopes with a large geographical distribution, ranging from Yangtze River to northeast China. *Phlebotomus sichuanensis*, a species closely related to *P. chinensis* in high altitude regions*,* has a long term disputation on its taxonomic status. Both species occur in the current epidemic regions and are responsible for the transmission of leishmaniasis*.* Population genetic analysis will help to understand the population structure and infer the relationship for morphologically indistinguishable cryptic species. In this study, microsatellite markers were used for studying the genetic differentiation between *P. chinensis* and *P. sichuanensis.*

**Methods:**

Sandflies were collected in 6 representative localities in China in 2005-2009. Ten microsatellite loci were used to estimate population genetic diversity. The intra-population genetic diversity, genetic differentiation and effective population size were estimated.

**Results:**

All 10 microsatellite loci were highly polymorphic across populations, with high allelic richness and heterozygosity. Hardy-Weinberg disequilibrium was found in 23 out of 60 (38.33%) comparisons associated with heterozygote deficits, which was likely caused by the presence of null allele and the Wahlund effect. Bayesian clustering analysis revealed three clusters. The cluster I included almost all specimens in the sample SCD collected at high altitude habitats in Sichuan. The other two clusters were shared by the remaining 5 populations, SCJ in Sichuan, GSZ in Gansu, SXL and SXX in Shaanxi and HNS in Henan. The diversity among these 5 populations was low (*F*_ST_ = -0.003-0.090) and no isolation by distance was detected. AMOVA analysis suggested that the variations were largely derived from individuals within populations and among individuals. Consistently, the analysis of ribosomal DNA second internal transcribed spacer (ITS2) sequence uncovered three types of variants, which corresponded with the three gene pools revealed by microsatellites.

**Conclusions:**

The data suggested that the SCD population carried a distinct gene pool, which was differentiated from the other populations. The high altitude ecological habitats, distinctive ITS2 and herein divergence inferred by microsatellite loci support the species status of *P. sichuanensis.* The *P. chinensis* populations did not have a significant divergence from each another.

## Background

Phlebotomine sandflies are small insects of great medical and veterinary relevance. In China, phlebotomine sandfly fauna includes over 40 species in five genera, *Phlebotomus* Rondani & Berte, *Sergentomyia* France & Farrot, *Idiophlebotomus* Quate & Fairchild, *Grassomyia* Theodor and *Chinius* Leng [1,2]. In the genus *Phlebotomus*, *P. chinensis* Newstead, *P. sichuanensis* Leng & Yin, *P. longiductus* Nitzulesc, *P. wuii* Yang & Xiong and *P. alexandri* Sinton are vectors of leishmaniasis [2]. Visceral leishmaniasis (VL) is caused by *Leishimania donovani*, which has been and still is a serious threat to public health in the endemic areas in China [3,4]. According to a report of VL surveillance in the period 2005 to 2010, most cases (97.7%) occurred in Xinjiang, Gansu and Sichuan [3].

**Figure 1:**
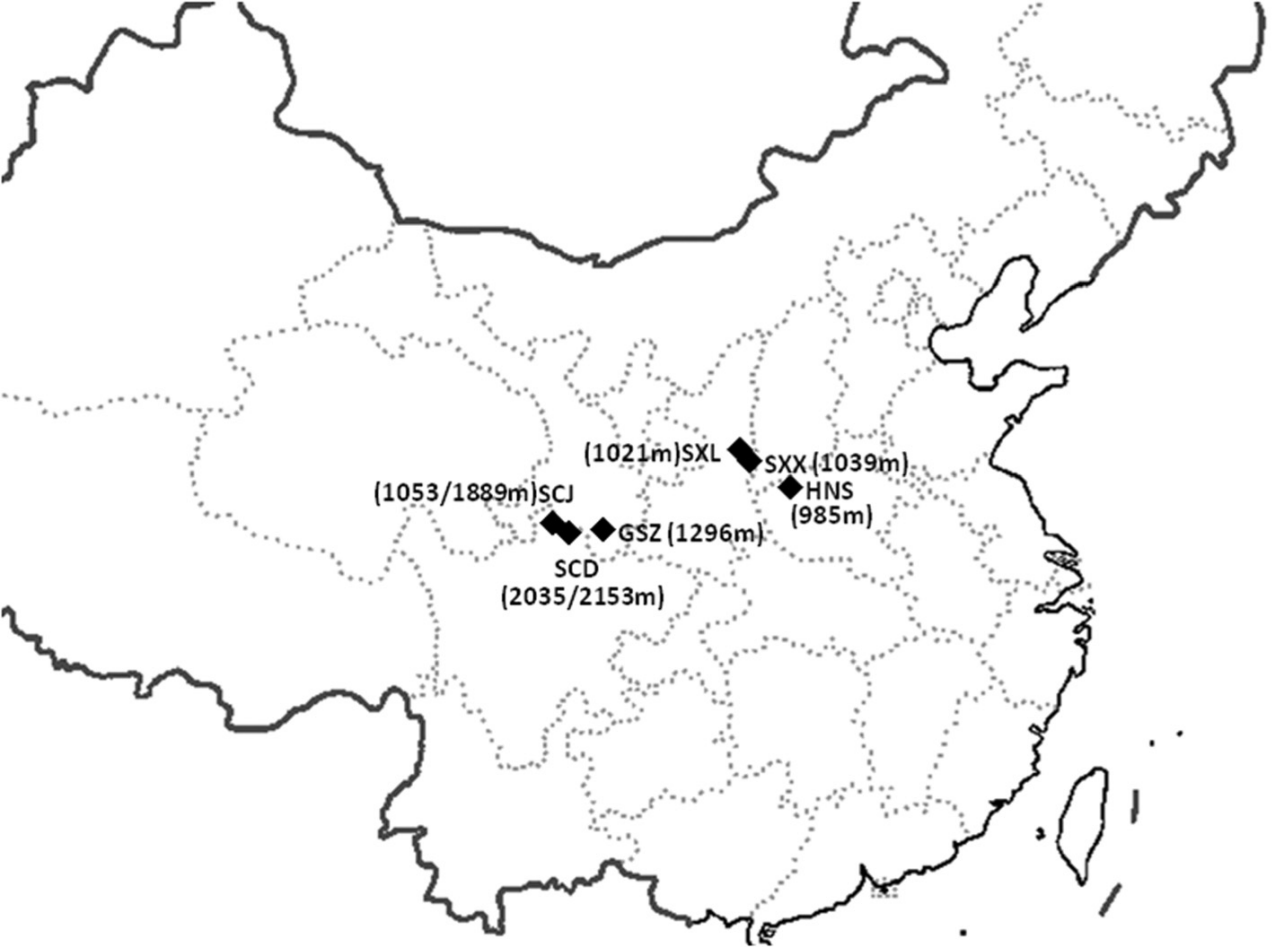
**Collection localities of *Phlebotomus *sandflies in China.** The diamond code indicates the field population. The number in the bracket is the altitude.

*Phlebotomus chinensis* is a prevalent species with wide geographical distribution, having records in 20 provinces ranging from Yangtze River to the northeast China [1]. In 1983, Leng and Yin described a new species, *P. sichuanensis*, based on the study of a large number of sandfly specimens that were collected in the area of 29-33°N, 102-106°E in Sichuan province in 1976-1980 [5]. The species was described based upon the morphological comparison with the specimens of *P. chinensis* collected in the type locality. Various morphometric and morphostructural characters were used for distinguishing *P. sichuanensis* from *P. chinensis*[5,6]. Male ascoid formula in *P. sichuanensis* is 2/3-8, 1/9-15, while it is 2/3-15 in *P. chinensis. Phlebotomus sichuanensis* is mainly distributed in the regions from 900 m to 2800 m in Gansu, Sichuan, Yunnan and Tibet [2,6,7] (see the map in Figure 1 in the ref. [6]). However, Xiong et al. (1990) argued that the morphological difference did not suffice to support *P. sichuanensis* as an independent species [8]. The body size of sandflies at high altitudes (above 2000 m) was larger than that of the sandflies at low altitudes (below 1600 m). The male ascoid formula 2/3-8, 1/9-15 was found in a high percentage (96.6%) in large size sandflies collected above 2000 m, and the formula 2/3-15 was found in small size sandflies collected at 1600 m. They considered the differences in body size and male ascoid formula as signs of different ecological types other than characters for taxonomic identification. Therefore, Xiong et al. (1990) referred to *P. sichuanensis* as a large type of *P. chinensis,* and synonymized *P. sichuanensis* to *P. chinensis*[8]*.* Epidemiologically, both sandflies are competent vectors of VL. Natural infection of *L. donovani* was detected in sandflies collected in high and low altitude regions [9]. In addition, sandflies from both collections were equally susceptible to *Leishmania* in experimental feeding on Chinese hamsters infected with *L. donovani*[8,9]. The vector importance of sandflies in leishmaniasis endemic areas urges the necessity to resolve the taxonomic dispute of *P. sichuanensis.* Apparently, non-morphological, convincing and distinctive taxonomic markers are needed for solving the identity issue.

Molecular markers, such as mitochondrial cytochrome b (Cyt b), ND1, ND4, the second internal transcribed spacer (ITS2) of ribosomal DNA and microsatellite markers, have been widely used for sandfly studies in taxonomic discrimination and inference of phylogenetic relationship [10-16], as well as population genetics studies [17-24]. In an effort to apply molecular data to address the taxonomic conflict of *P. sichuanensis*, we conducted six sandfly collections in 2005-2009, where *P. chinensis* and *P. sichuanensis* occur. Among them, sample SCD was collected in habitats at high altitudes (above 2000 m), a typical locality of *P. sichuanensis*. Previously we have reported the sequence variation of the ITS2 and Cyt b generated from these samples [25]. Based on the ITS2 and Cyt b variations, *P. chinensis* was clearly separated from *P. longiductus, P. perfiliewi, P. pernicious, P. ariasis, P. duboscqi* and *P. alexandri*. The partial ITS2 sequences of SCD specimens showed distinctive variation from that of *P. chinensis*[25].

Microsatellites are highly polymorphic genetic markers that evolve much faster than mitochondrial or nuclear genes, which are particularly useful for resolving the structure of populations at a finer geographical and evolutionary scale. In this study, we conducted population genetic analysis of the same six sandfly collections reported in the paper above [25], based on 10 microsatellite loci. The population genetic analysis would help to understand the population structure and infer the relationship for morphologically indistinguishable cryptic species.

## Methods

### Sandfly collections and species identification

There are four species in the subgenus *Adlerius* in China, *P. chinensis, P. sichuanensis, P. longiductus* and *P. fengi* Leng & Zhang. The distribution region of *P. longiductus* and *P. fengi* is limited to Xingjiang and Yunnan. In this study, wild adult sandflies were collected in 2005 and 2009 from six locations in the range from 104°15′N to 110°60′ N, and 33°18′E to 36°18′ E in Sichuan, Gansu, Shaanxi, Henan provinces (Table 1, Figure 1). These collection sites corresponded to the current epidemic regions of leishmaniasis (see the VL distribution map in Figure 1 in ref. [3]). The sandfly specimens were collected by using human landing catches at livestock corrals or mountain caves, under the NSFC ethical guidelines for biomedical research involving living animals and human subjects. The collections in livestock corrals were conducted with the consent of the owners. The specimens were kept individually in tubes filled with silica gel at 4°C until dissection was performed. The specimens from the six populations were identified as *P. chinensis* sensu lato based on the morphological characters of the female or male external genitalia, the structure of the pharyngeal armature and the spermatheca [26] (see Results).

**Table 1:** Sandflies collections in China

**Code**	**Collection site**	**No. of specimens (♀: ♂)**	**Date**	**Latitude/longitude coordinates**	**Altitude (m)**
HNS	Shanxian, Henan	15: 12	7/05	110°10′N/34°24′E	985
SCJ	Yongfeng Jiuzhaigou, Sichuan	16: 12	7/09	104°15′N/33°14′E	1503/1889
SCD	Dongshan Jiuzhaigou, Sichuan	4: 22	7/09	104°15′N/33°14′E	2035/2153
SXX	Xiataitou Yichuan, Shaanxi	24: 1	7/09	110°37′N/36°18′E	1039
SXL	Luodong Yichuan, Shaanxi	23: 3	7/09	110°60′N/36°10′E	1021
GSZ	Wenxian, Gansu	11: 15	7/09	104°25′E/33°18′N	1296

### Microsatellite genotyping

Following morphological identification, the genomic DNA was extracted from individual adults from dissected tissue using the Insect Tissue DNA Extraction Kit (Biyuntian, Jiangsu China), using the manufacturer’s protocol. Ten microsatellite loci [27], GA1 (GenBank accession number FJ919882), GA5 (FJ919907), GA13 (FJ919884), GA24 (FJ919885), GA63 (FJ919932), GA76 (FJ919890), GA109 (FJ919900), AAT3 (FJ919917), HN54 (FJ919923) and TG1 (FJ919894), were used for genotyping. Microsatellite loci were amplified according to the detailed protocols described by Schuelke [28]. Each PCR reaction contained three primers: a sequence-specific forward primer with a M13(-21) adapter (5′- TGT AAA ACG ACG GCC AGT -3′) conjugated at its 5′ end, a sequence-specific reverse primer, and a universal FAM-labeled M13(-21) primer. Amplified fragments were separated by capillary electrophoresis in an automatic sequencer (ABI 3770, Applied Biosystems, Foster City, CA) and size was scored using GENOTYPER 3.7 software (Applied Biosystems, Foster City, CA).

### Data analysis

Genetic diversity within samples and overall was measured at each locus by estimates of number of alleles *A,* allele richness *Rs*, inbreeding coefficient *F*_IS_, and observed heterozygosity *H*o [29], using the software FSTAT 2.9.3.2 [30]. Within each locality the frequency of null alleles was determined using the Brookfield 2 estimate [31], and the allele and genotype frequencies were then adjusted accordingly by using MICRO-CHECKER 2.2.3 [32]. To determine if the null alleles impacted the population genetic analyses, we performed these analyses both before and after the dataset were adjusted for estimated null allele frequencies. This treatment did not significantly change the degree or statistical significance of the estimated parameters. Genotypic frequencies were tested against Hardy-Weinberg equilibrium (HWE) for each locus in pooled populations and in each sample. Statistical significance was assessed by the exact probability test available in GENEPOP 3.2 [33]. Linkage disequilibrium (LD) between loci was tested by exact tests on contingency tables, also available in GENEPOP.

A Bayesian approach was used to infer the number of clusters (*K*) in the data set without prior information of the sampling locations, implemented with STRUCTURE 2.2 [34]. A model where the allele frequencies were correlated within populations was assumed (λ was set at 1, the default value). The software was run with the option of admixture, allowing for some mixed ancestry within individuals, and α was allowed to vary. Twenty independent runs were carried out for each value of *K* (*K* = 1 to 6), with a burn-in period of 1,000,000 chains and 1,000,000 Markov chains Monte Carlo replications. The method of Evanno was used to determine the most likely number of clusters [35]. This approach uses an *ad hoc* quantity, Δ*K*, based on the second order rate of change of the likelihood function between successive values of *K*.

Genetic differentiation was estimated by calculating *F*_ST_ between pairs of populations using ARLEQUIN 3.0 [36] and GENEPOP [33]. The number of migrants per population per generation (*N*m) between localities was estimated from pairwise *F*_ST_[37]. An analysis of molecular variance (AMOVA) was used to examine the distribution of genetic variation in ARLEQUIN using *F*_ST_. We focused on estimates of *F*_ST_ performed under the infinite alleles model (IAM) because this model is considered more reliable when fewer than 20 microsatellites are used [38]. The significance for all calculations was assessed by 10,000 permutations and the *P*-values. The isolation by distance model was investigated as a potential explanation for the observed population differentiation. The significance of the regression of genetic differences on geographic distance between sample pairs was tested using a Mantel test [39] with 100,000 permutations in GENEPOP.

The long-term effective population size (*Ne*) was estimated using NEESTIMATOR 1.3 [40] based on the heterozygote excess and linkage disequilibrium models.

## Results

### Species identification

Wild sandflies were collected at 6 locations in China (Figure 1), a total of 158 specimens including 93 females and 65 males were used in the study (Table 1). In these locations, the only documented species in the subgenus *Adlerius* were *P. chinensis* and *P. sichuanensis*[5,6,26]. The morphologic characters of *P. chinensis* were as follows: transverse ridges on the pharyngeal armature, ellipsoid shape to spermatheca, number of spermatheca segments 13 or 14, length of spermatheca duct longer than 2.5 times spermatheca, and the length ratio of genital filament to pompetta 1 : 5.6. Based on the above key criteria, all 158 specimens were identified as *P. chinensis* s. l. There are no absolute morphological characters to distinguish *P. chinensis* with *P. sichuanensis,* however, it was noted that the morphology of 26 specimens in the SCD population conformed to that of *P. sichuanensis* as described in [5,6].

### Genetic variability within populations

Ten microsatellite loci were genotyped for these specimens. The number of alleles (*A*) per locus ranged from 2.3 at locus *HN54* to 9.83 at locus *GA76*. As an exception, only one allele was detected at locus *GA1* in sample GSZ (Table 2). The minimum mean number of alleles of all loci was 5.5 in sample SXL, and the maximum was 7.1 in sample HNS. The average allelic richness per locus (*Rs*) across 6 samples was 3.72, ranging from 3.145 in SCD to 3.849 in HNS. The average observed heterozygosity (*H*_O_) across all samples ranged from 0.236 (*TG1*) to 0.77 (*GA109*), the minimum was in SXL (0.375), the maximum in HNS (0.503). The mean expected heterozygosity (*H*_E_) was 0.619, with a range of from 0.49 (SCD) to 0.693 (HNS). Putting these statistics together, the HNS population showed the highest diversity, whereas the SCD population showed the lowest diversity.

**Table 2:** Summary of microsatellite variation at 10 loci in this study

**Samples**		**GA1**	**GA5**	**GA13**	**GA24**	**GA63**	**GA76**	**GA109**	**AAT3**	**HN54**	**TG1**	**All loci**
SXL (N = 26)	*A*	2	5	8	6	6	11	6	5	3	3	5.500
	*R*_*S*_	1.400	3.341	4.765	3.656	3.726	4.540	3.389	3.176	1.918	1.622	3.153
	*H*_E_	0.108	0.694	0.836	0.736	0.724	0.782	0.701	0.614	0.298	0.160	0.565
	*H*_O_	0.000	0.539	0.500	0.423	0.286	0.400	0.692	0.400	0.346	0.167	0.375
	*r*	0.095	0.085	0.176	0.136	0.247	0.203	0.000	0.126	0.654	0.000	0.181
	*F*_IS_	1.000	0.227	0.406	0.430*	0.611*	0.497*	0.012*	0.353*	-0.166	-0.041	0.342
SXX (N = 25)	*A*	5	5	8	6	5	13	5	5	2	5	5.900
	*R*_*S*_	2.875	3.005	4.632	3.711	2.993	5.450	3.369	3.179	1.857	1.950	3.302
	*H*_E_	0.482	0.632	0.809	0.746	0.637	0.868	0.698	0.657	0.327	0.235	0.609
	*H*_O_	0.357	0.440	0.696	0.600	0.267	0.684	0.640	0.520	0.400	0.167	0.477
	*r*	0.073	0.111	0.053	0.033	0.216	0.087	0.003	0.075	0.600	0.052	0.132
	*F*_IS_	0.266	0.308	0.143	0.199	0.590	0.216	0.085*	0.212	-0.231	0.295	0.221
HNS (N = 27)	*A*	8	6	6	5	9	13	7	6	3	8	7.100
	*R*_*S*_	3.230	3.366	4.178	3.615	5.014	5.556	3.411	3.889	1.918	4.317	3.849
	*H*_E_	0.516	0.667	0.776	0.736	0.854	0.873	0.708	0.721	0.298	0.777	0.693
	*H*_O_	0.444	0.440	0.522	0.478	0.389	0.739	0.704	0.667	0.346	0.300	0.503
	*r*	0.038	0.129	0.135	0.119	0.241	0.062	0.000	0.000	0.654	0.260	0.167
	*F*_IS_	0.142	0.345	0.332	0.356	0.552*	0.156	0.006*	0.077*	-0.166	0.620*	0.279
GSZ (N = 26)	*A*	1	4	9	7	6	9	7	7	2	6	5.800
	*R*_*S*_	1.000	2.900	5.054	4.121	4.001	4.469	3.596	4.560	1.870	3.669	3.524
	*H*_E_	0.000	0.618	0.855	0.781	0.772	0.759	0.706	0.819	0.337	0.717	0.636
	*H*_O_	0.000	0.333	0.462	0.692	0.250	0.467	0.920	0.381	0.417	0.412	0.433
	*r*	NA	0.169	0.233	0.000	0.287	0.154	0.000	0.233	0.583	0.167	0.203
	*F*_IS_	NA	0.466	0.465*	0.116	0.682*	0.393*	-0.310*	0.541*	-0.243	0.433*	0.282
SCJ (N = 28)	*A*	7	4	7	9	9	5	9	5	2	6	6.300
	*R*_*S*_	3.130	2.649	3.913	3.899	4.368	5.000	3.490	2.891	1.857	3.216	3.441
	*H*_E_	0.540	0.474	0.736	0.728	0.775	0.893	0.694	0.542	0.308	0.690	0.638
	*H*_O_	0.214	0.440	0.393	0.500	0.482	0.250	0.929	0.360	0.370	0.364	0.430
	*r*	0.281	0.017	0.192	0.125	0.194	0.200	0.000	0.112	0.630	0.164	0.191
	*F*_IS_	0.511*	0.051	0.423*	0.332	0.394*	0.750	-0.344*	0.321	-0.231	0.550*	0.328
SCD (N = 26)	*A*	9	7	8	5	6	8	4	5	2	2	5.600
	*R*_*S*_	4.553	3.228	3.991	2.631	2.839	4.764	2.849	3.051	1.268	2.279	3.145
	*H*_E_	0.705	0.548	0.652	0.420	0.426	0.761	0.490	0.458	0.071	0.374	0.490
	*H*_O_	0.692	0.720	0.667	0.500	0.308	0.546	0.720	0.320	0.000	0.000	0.447
	*r*	0.022	0.000	0.019	0.000	0.104	0.136	0.000	0.111	0.981	0.332	0.171
	*F*_IS_	0.217	-0.059	0.182	-0.007	0.390	0.351	-0.295	0.467*	1.000	0.871*	0.244
All samples (N = 158)	*A*	5.330	5.170	7.670	6.330	6.830	9.830	6.330	5.500	2.300	5.170	6.046
	*R*_*S*_	2.801	3.398	5.107	3.688	4.006	5.851	3.466	3.720	2.005	3.166	3.721
	*H*_E_	0.394	0.624	0.792	0.707	0.7160	0.833	0.680	0.656	0.276	0.515	0.619
	*H*_O_	0.275	0.483	0.536	0.533	0.332	0.514	0.770	0.441	0.318	0.236	0.447
	*r*	0.102	0.085	0.135	0.069	0.215	0.140	0.001	0.109	0.684	0.162	0.174
	*F*_IS_	1.618	1.723	2.492	1.959	2.097	2.988	1.945	1.860	0.983	1.660	1.932

First column indicates collection sites and sample size in parenthesis; *A,* number of alleles; *R*_*S*_*,* allelic richness; *H*_E_*,* expected heterozygosity; *H*_O_*,* observed heterozygosity; *r*: frequency of null alleles; *F*_IS_, inbreeding coefficient; All loci/samples, mean values over loci or populations; NA, no data; *, Probability test against Hardy- Weinberg proportions (*P* < 0.001).

The Hardy-Weinberg exact tests were performed for the 10 loci. No locus was in HWE for all the samples assayed, except HN54. At the population level, 23 out of 60 (38.33%) comparisons did not conform to Hardy-Weinberg expectations, and the deviations were associated with positive inbreeding coefficient (*F*_IS_), reflecting heterozygosity deficits (Table 2). Significant deviation from HWE varied across loci in a population-dependent manner. The GSZ population had the highest number of loci in departure from HWE (6 of 10), while the SXX population had the fewest (1 of 10) (Table 2). In all samples, some specimens failed to amplify at one locus while they succeeded to amplify at the remaining loci, suggesting the presence of null alleles. Estimates of the frequency of null alleles are given in Table 2. The highest *r* value was at the locus *HN54*.

Fisher’s exact tests were conducted for LD within each of the six populations. Out of 270 comparisons only 17 pairs (6.30%) were at LD (*P* < 0.01). The pair of *GA10* and *HN54* appeared at LD in all populations, except in the SCD population.

### Genetic differentiation among populations

**Figure 2:**
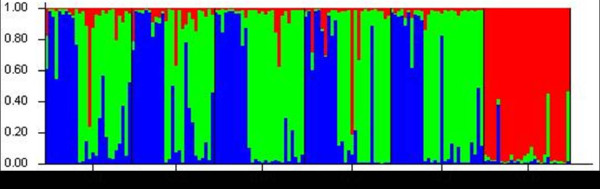
**Bayesian cluster analysis using STUCTURE.** Graphical representation of the data set for the most likely *K* (*K* = 3), where each color corresponds to a suggested cluster and each individual is represented by a vertical bar. The X-axis corresponds to population codes. The Y-axis presents the probability of assignment of an individual to each cluster.

**Figure 3:**
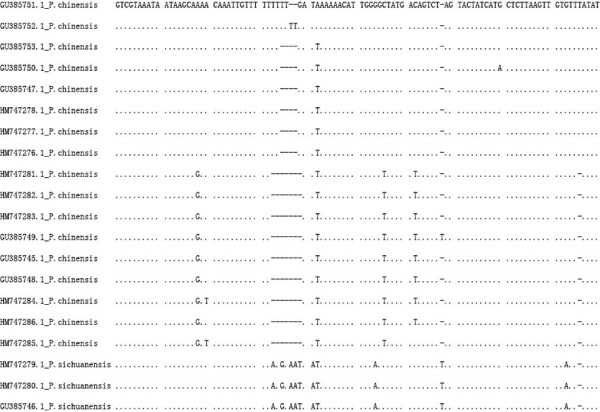
**The ITS2 sequence alignment of *P. chinensis *and *P. sichuanensis*.** Presented partial sequences show the variations. There are three major types of variants.

**Figure 4:**
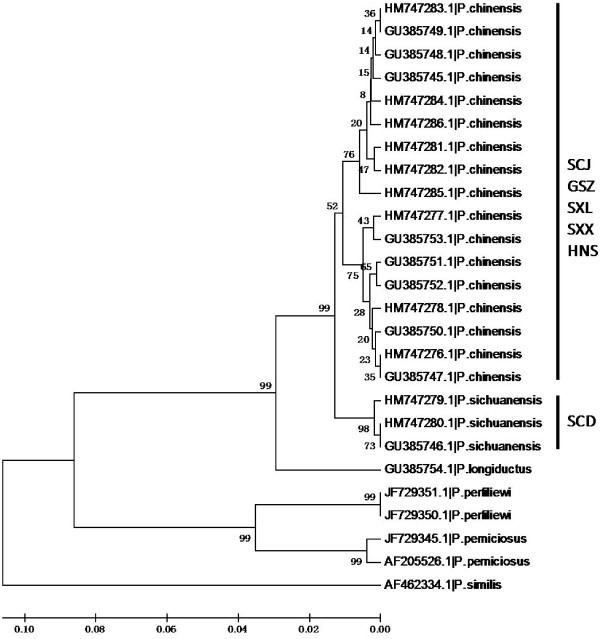
**The UPGMA tree of *Phlebotomus *sandflies inferred by rDNA-ITS2 sequences.** The phylogram was generated using MEGA 5.10. The bootstrap values (1000 replications) are shown on the branch. The sequence id is presented by the Genbank accession numbers followed by the species identity. Population id is indicated next to the clades.

The significant deviations from HWE with heterozygote deficiency and the presence of LD suggest the presence of population subdivision within samples (Wahlund effect). We therefore examined if there were different gene pools in these samples. The Bayesian cluster analysis divided specimens into three gene clusters (posterior probability of Bayesian clustering Ln (D) likelihood score optimal for *K* = 3 clusters) (Figure 2). Sample SCD was clearly distinct from the others; almost all individuals belonged to cluster I (red). The rest of the five samples were mixed with the individuals from the other two gene pools. This pattern was consistent with the pattern inferred from ITS2 sequence comparison [25]. ITS2 sequences of those individuals showed three major types of variants. Based on the alignment of ITS2 sequences (Figure 3), phylogenetic analysis separated those variants into three clades (Figure 4). The SCD collection formed a single clade, the other five collections were clustered into one clade with two sister branches. Interestingly, each of these five collections was mixed with the two major types of ITS variants. In the analysis we also included the ITS2 sequences (PopSet 290794958) available in the NCBI deposited by Gu and Zhang. These specimens were collected in Dongshan, Sichuan; Wen County, Gansu; Sanmenxia, Henan; Yichuan, Shaanxi. Intriguingly, the three major types of ITS2 sequences were found in the collection (Figure 3). The sequence GU385746 was from a specimen collected in Dongshan, Sichuan, where the SCD was sampled in this study. The independent data added a strong support for the presence of distinct genetic clusters in the sandflies.

The gene flow and *F*_ST_ were estimated in a location based manner for the six samples, which is presented in Table 3. The SCD population diverged from the other five populations. The pairwise *F*_ST_ value was greater and the *N*m value was lower between SCD and the others than the pairwise comparisons among the other five populations. Therefore, the SCD represented a population diverged from the other five *P. chinensis* populations in the study. Tests of isolation by distance were performed for five populations except SCD. No statistically significant correlations were detected between genetic differentiation and geographic distances based on the Mantel test (*R*^2^=0.2846, *P*=0.10). The results suggested that geographic distance did not significantly contribute to the genetic differentiation observed in the *P. chinensis* populations.

**Table 3:** **Pairwise genetic distance (*F***_**ST**_**) and gene flow ( *N *m) for this study populations**

**Population**	**SXL**	**SXX**	**HNS**	**GSZ**	**SCJ**	**SCD**
SXL		- (10)	4.354(175)	5.245(600)	3.924(620)	1.081
SXX	-0.003*		4.991(170)	4.388(595)	2.516(615)	1.169
HNS	0.054*	0.048*		9.478(625)	4.296(663)	1.166
GSZ	**0.046**	0.054*	0.026*		6.581(50)	1.419
SCJ	0.060*	0.090*	**0.055**	**0.037**		0.998
SCD	**0.188**	**0.176**	**0.177**	**0.150**	**0.200**	

The pairwise *N*m values are above diagonal; pairwise *F*_ST_ values below diagonal and within population along the diagonal. Approximate geographical distances in km are in parentheses. Abbreviations of localities are shown in Table 1. * *P* < 0.05.

In the hierarchical AMOVA, both the “among individuals within populations” and “among individuals” variance components were considerably high (83.35%), suggesting that the total variances largely came from individual variations (Table 4).

**Table 4:** AMOVA analysis of genetic variation in this study

**Source of variation**	**Sum of squares**	**Variance components**	**Percentage of variation (%)**
Among groups	45.426	0.443	12.02
Among populations within groups	43.835	0.171	4.63
Among individuals within populations	494.072	0.835	22.65
Within individuals	305.500	2.239	60.70

### Effective population size

Estimates of long-term *Ne* varied considerably depending on the model used. Under the heterozygote excess model, all of the *Ne* estimates were infinity. Under the linkage disequilibrium model, diverse *Ne* values were detected across populations (Table 5). The six populations showed variability of *Ne,* from 5.7 (SXL) to 264.4 (SCJ).

**Table 5:** **Estimated *Ne *based on the linkage disequilibrium (LD) model in this study**

**Population**	***Ne***	**95% CI**
SXL	5.7	4.9-6.6
SXX	10.5	8.7-13.0
HNS	17.1	13.9-21.8
GSZ	12.4	10.0-15.9
SCJ	264.4	46.9-∞
SCD	10.1	8.1-13.1

CI: confidence intervals; ∞: infinity.

## Discussion

*Phlebotomus chinensis* s. l. is the major vector for VL transmission in the endemic regions except Xinjiang in China*.* The sandflies in these regions have two major types of ecological habitats. In the open flatland regions with an altitude <900 m, such as the vast plain area north of the Yangtze River, the sandflies are found in the indoor shelters such as human dwellings and domestic animal sheds. The transmission of VL is largely from human to human. In the mountain areas, the sandflies are largely exophilic, breeding in a variety of wild habitats, such as various caves and rodent burrows. If human dwellings are available, the sandflies feed on humans or domestic animals. The dogs are the main animal hosts of *L. donovani*, and VL is largely transmitted between dogs and humans. In the areas with an altitude >2000 m, usually there are no human dwellings. The sandflies feed on wild animals for breeding, and transmit *Leishmania* among wild animals [3,41]. *Phlebotomus sichuanensis*[5,6]*,* or a large type of *P. chinensis*[8,42] were mostly found in the high altitude areas in Sichuan, Yunnan, Shaanxi, Gansu and Tibet [6]. The current epidemiological status of leishmaniasis in China was reviewed recently. In brief, most cases of leishmaniasis occurred in Xingjiang, Gansu and Sichuan [3]. In past decades, the density of sandflies dropped drastically, most likely due to the wide applications of insecticides and environmental changes. In this study, 6 collection sites were chosen in the Loess Plateau, hills and mountainous areas with an altitude of 985-2153 m, where mountainous sub-type zoonotic VL occurs (see Figure 1 in the ref. [3]). The sampling sites had a representative geographic coverage of *P. chinensis* and *P. sichuanensis* in the high altitude areas.

The significant deviations from HWE due to the heterozygote deficits were detected in most samples, suggesting the presence of population subdivision within samples (Wahlund effect). Indeed, cluster analysis, implemented by using STRUCTURE, recognized three gene clusters in the six sandfly collections. Almost all individuals in SCD population were assigned to cluster I (Figure 2). The data identified SCD as a distinct genetic unit with an isolated gene pool. The other two genetic clusters were distributed in the rest of five collections. Consistent with the pattern inferred by microsatellite, the ITS2 sequences from the SCD (GenBank accession HM747279, HM747280) plus one sequence (GU385746) from the Gu and Zhang collection were clustered in one clade, while the other ITS2 sequences grouped into two sister clades (Figure 4). Therefore, the ITS2 analysis supported the genetic distinction between SCD and other *P. chinensis* collections. When six sample collections were compared, the differentiation between SCD and others was evident by high *F*_ST_ and reduced gene flow (*F*_ST_ = 0.15-0.20, *N*m *=* 0.998-1.419, Table 3). Apparently, SCD represents a genetic unit that has diverged from the other populations. The SCD specimens were sampled in a location at an elevation of 2035-2153 m, a typical habitat of *P. sichuanensis*[6]. The morphological characters of the SCD population specimens were consistent with the descriptions of *P. sichuanensis*[5,6], although morphology only is often not conclusive for distinguishing the two species*.* Taken together, both ITS2 and microsatellites separated SCD from other populations. Therefore, we concluded that the molecular data supported the species status of *P. sichuanensis.* The ecological adaptation of *P. sichuanensis* in high altitude regions may have contributed to its separation from *P. chinensis* in the low altitude regions. The relative low level of divergence may represent adaptation-driven incipient speciation of *P. sichuanensis* by divergent environment at high altitudes. Such ongoing speciation has been exemplified in the mosquito *Anopheles gambiae*, in which two molecular forms, M and S, are experiencing a speciation-with-ongoing-gene-flow [43-45]. Further investigation with more genetic markers, eventually whole genomic sequencing, will clarify the issue ultimately.

The pairwise level of genetic variation was small in the remaining five populations (*F*_ST_ = -0.003-0.090, *N*m *=* 2.516-6.581, Table 3). The two gene clusters did not show any correlation to geographic origin. The AMOVA data suggested that most variations among populations may attribute to the individual variation, which may explain the source of the two clusters that were shared by the *P. chinensis* populations. The small genetic differentiation in *P. chinensis* populations and no evidence of isolation by distance suggested that no obvious barriers limit the dispersal of sandflies in the *P. chinensis* populations sampled in this study.

Leishmaniasis has shown an increasing trend in recent years in China [3]. Different *Leishmania* genotypes have been found in different geographic origins, such as hill, plain and desert [46]. The current study investigated the sandflies in the mountainous regions where the VL is epidemic. Both *P. chinensis* and *P. sichuanensis* are competent vectors [8,9]. The different ecological habitats may be accompanied by different behavior and physiology that may affect disease transmission and compromise anti-vector measures. Particularly, *P. sichuanensis* occurs in regions of above 2000 m, where *Leishmania* has been cycling among wild reservoir animal hosts. It is conceivable that *P. sichuanensis* constitutes a necessary component connecting the zoonotic VL to the human community. The current characterization of sandfly population structure and ITS2 sequences provide molecular data to develop objective and reliable methods for molecular identification of sandfly specimens. Such tools will be particularly useful to further investigate the ecology, behavior and vector capacity of *P. chinensis* and *P. sichuanensis* in the mountainous regions. This type of data will facilitate the development of appropriate measures to control sandfly vectors.

## Conclusion

The microsatellite data suggest that SCD represents a population of *P. sichuanensis* possessing a distinct gene pool, which was differentiated from the *P. chinensis* populations. The molecular data support the species status of *P. sichuanensis.* The five *P. chinensis* populations did not have a significant divergence from each other. The genetic distinction of *P. sichuanensis* from *P. chinensis* warrants further study to explore potential influence on physiology, behavior, and vector competence that may be associated with different ecological habitats between the two species.

## Competing interests

The authors declare that they have no competing interests.

## Authors’ contributions

YM conceived and designed the experiments. LZ performed the experiments. YM and JX analyzed the data, wrote the manuscript. All authors read and approved the final manuscript.

## References

[B1] Leng YJ Zhang LM Check list and geographical distribution of phlebotomine sandflies in China Ann Trop Med Parasitol 1993 87 1 83 94 8346993 10.1080/00034983.1993.11812740

[B2] Zhang LM Leng YJ Eighty-year research of phlebotomine sandflies (Diptera: Psychodidae) in China (1915-1995). II. Phlebotomine vectors of leishmaniasis in China Parasite 1997 4 4 299 306 9587598 10.1051/parasite/1997044299

[B3] Wang JY Cui G Chen HT Zhou XN Gao CH Yang YT Current epidemiological profile and features of visceral leishmaniasis in People’s Republic of China Parasit Vectors 2012 5 31 10.1186/1756-3305-5-31 22316234 PMC3311609

[B4] Guan LR Shen WX Recent advances in visceral leishmaniasis in China Southeast Asian J Trop Med Public Health 1991 22 3 291 298 1818378

[B5] Leng YJ Yin ZC The taxonomy of phlebotomine sandflies (Diptera: Psychodidae) of Sichuan Province, China, with descriptions of two species, *Phlebotomus (Adlerius) sichuanensis* sp. n. and *Sergentomyia (Neophlebotomus) zhengjiani* sp. n Ann Trop Med Parasitol 1983 77 4 421 431 6639187 10.1080/00034983.1983.11811731

[B6] Leng YJ Zhang LM Chinese phlebotomine sandflies of subgenus Adlerius nitzulescu,(Diptera: Psychodidae) and the identity of *Phlebotomus sichuanensis* Leng & Yin, 1983. Part I-Taxonomical study and geographical distribution Parasite 1931 8 1 3 9 11304947 10.1051/parasite/2001081003

[B7] Leng YJ Zhang LM Zhang BX A study of phlebotomine sandflies (Diptera: Psychodidae) in Yunnan Province, China. I. Phlebotomine sandflies of northeastern and western Yunnan Parassitologia 1991 33 Suppl 373 375 1841230

[B8] Xiong GH Jin CF Hong YM Further study on the type of sandfly *Ph. chinensis* in southern Gansu and Northern Sichuan Endemic Dis Bull 1990 5 53 60

[B9] Xiong GH Jin CF Studies on the longitudinal distribution of sandfly *Phlebotomus chinensis* and its relation to Kala Azar in southern Gansu and northern Sichuan Endemic Diseases Bulletin 1989 4 4 15 21

[B10] Latrofa MS Annoscia G Dantas-Torres F Traversa D Otranto D Towards a rapid molecular identification of the common phlebotomine sand flies in the Mediterranean region Vet Parasitol 2012 184 2–4 267 270 21924834 10.1016/j.vetpar.2011.08.031

[B11] Kumar NP Srinivasan R Jambulingam P DNA barcoding for identification of sand flies (Diptera: Psychodidae) in India Mol Ecol Resour 2012 12 3 414 420 10.1111/j.1755-0998.2012.03117.x 22277023

[B12] Khalid N Elnaiem D Aboud M Al Rabba F Tripet F Morphometric and molecular differentiation of *Phlebotomus (Phlebotomus)* sandflies Med Vet Entomol 2010 4 4 352 360 20633225 10.1111/j.1365-2915.2010.00893.x

[B13] Depaquit J Naucke TJ Schmitt C Ferte H Leger N A molecular analysis of the subgenus *Transphlebotomus* Artemiev, 1984 (Phlebotomus, Diptera, Psychodidae) inferred from ND4 mtDNA with new northern records of *Phlebotomus mascittii* Grassi, 1908 Parasitol Res 2005 95 2 113 116 10.1007/s00436-004-1254-x 15666186

[B14] Depaquit J Ferte H Leger N Lefranc F Alves-Pires C Hanafi H Maroli M Morillas-Marquez F Rioux JA Svobodova M ITS2 sequences heterogeneity in *Phlebotomus sergenti* and *Phlebotomus similis* (Diptera, Psychodidae): possible consequences in their ability to transmit *Leishmania tropica* Int J Parasitol 2002 32 9 1123 1131 10.1016/S0020-7519(02)00088-7 12117495

[B15] Depaquit J Ferte H Leger N Killick-Kendrick R Rioux JA Killick-Kendrick M Hanafi HA Gobert S Molecular systematics of the phlebotomine sandflies of the subgenus *Paraphlebotomus* (Diptera, Psychodidae, Phlebotomus) based on ITS2 rDNA sequences. Hypotheses of dispersion and speciation. Insect Mol Biol 2000 9 3 293 300 10.1046/j.1365-2583.2000.00179.x 10886413

[B16] Di Muccio T Marinucci M Frusteri L Maroli M Pesson B Gramiccia M Phylogenetic analysis of *Phlebotomus* species belonging to the subgenus *Larroussius* (Diptera, psychodidae) by ITS2 rDNA sequences Insect Biochem Mol Biol 2000 30 5 387 393 10.1016/S0965-1748(00)00012-6 10745162

[B17] Belen A Kucukyildirim S Alten B Genetic structures of sand fly (Diptera: Psychodidae) populations in a leishmaniasis endemic region of Turkey J Vector Ecol 2011 36 Suppl 1 S32 S48 21366779 10.1111/j.1948-7134.2011.00110.x

[B18] Hamarsheh O Presber W Yaghoobi-Ershadi MR Amro A Al-Jawabreh A Sawalha S Al-Lahem A Das ML Guernaoui S Seridi N Population structure and geographical subdivision of the *Leishmania major* vector *Phlebotomus papatasi* as revealed by microsatellite variation Med Vet Entomol 2009 23 1 69 77 10.1111/j.1365-2915.2008.00784.x 19239616

[B19] Hamarsheh O Presber W Al-Jawabreh A Abdeen Z Amro A Schonian G Molecular markers for *Phlebotomus papatasi* (Diptera: Psychodidae) and their usefulness for population genetic analysis Trans R Soc Trop Med Hyg 2009 103 11 1085 1086 10.1016/j.trstmh.2009.02.011 19303124

[B20] Depaquit J Lienard E Verzeaux-Griffon A Ferte H Bounamous A Gantier JC Hanafi HA Jacobson RL Maroli M Moin-Vaziri V Molecular homogeneity in diverse geographical populations of *Phlebotomus papatasi* (Diptera, Psychodidae) inferred from ND4 mtDNA and ITS2 rDNA epidemiological consequences Infect Genet Evol 2008 8 2 159 170 10.1016/j.meegid.2007.12.001 18243814

[B21] Hamarsheh O Presber W Abdeen Z Sawalha S Al-Lahem A Schonian G Genetic structure of mediterranean populations of the sandfly *Phlebotomus papatasi* by mitochondrial cytochrome b haplotype analysis Med Vet Entomol 2007 21 3 270 277 10.1111/j.1365-2915.2007.00695.x 17897368

[B22] Hamarsheh O Amro A Characterization of simple sequence repeats (SSRs) from *Phlebotomus papatasi* (Diptera: Psychodidae) expressed sequence tags (ESTs) Parasit Vectors 2011 4 189 10.1186/1756-3305-4-189 21958493 PMC3191335

[B23] Aransay AM Ready PD Morillas-Marquez F Population differentiation of *Phlebotomus perniciosus* in Spain following postglacial dispersal Heredity (Edinb) 2003 90 4 316 325 10.1038/sj.hdy.6800246 12692585

[B24] Mahamdallie SS Pesson B Ready PD Multiple genetic divergences and population expansions of a Mediterranean sandfly, *Phlebotomus ariasi*, in Europe during the Pleistocene glacial cycles Heredity (Edinb) 2011 106 5 714 726 10.1038/hdy.2010.111 20736970 PMC3186226

[B25] Zhang L Ma Y Identification of *Phlebotomus chinensis* (Diptera: Psychodidae) inferred by morphological characters and molecular markers Entomotaxonomia 2012 34 1 71 80

[B26] Lu B Wu H Classification and identification of important medical insects of China 2003 Henan: Henan Science and Technology Publishing House

[B27] Zhang L Ma Y Isolation of microsatellite DNA and the polymorphic locus screening from *Phlebotomus chinensis* Chin J Parasitol Parasit Dis 2009 27 6 503 507 20232635

[B28] Schuelke M An economic method for the fluorescent labeling of PCR fragments Nat Biotechnol 2000 18 2 233 234 10.1038/72708 10657137

[B29] Nei M Molecular evolutionary genetics 1987 New York: Colombia University Press

[B30] Goudet J FSTAT (Version 1.2): A computer program to calculate F-statistics J Hered 1995 86 6 485 486

[B31] Brookfield JF A simple new method for estimating null allele frequency from heterozygote deficiency Mol Ecol 1996 5 3 453 455 8688964 10.1111/j.1365-294x.1996.tb00336.x

[B32] Van Oosterhout C Hutchinson WF Wills DPM Shipley P Micro-Checker: software for identifying and correcting genotyping errors in microsatellite data Mol Ecol Notes 2004 4 3 535 538 10.1111/j.1471-8286.2004.00684.x

[B33] Raymond M Rousset F GENEPOP (Version 1.2): Population genetics software for exact tests and ecumenicism J Hered 1995 86 3 248 249

[B34] Evanno G Regnaut S Goudet J Detecting the number of clusters of individuals using the software STRUCTURE: a simulation study Mol Ecol 2005 14 8 2611 2620 10.1111/j.1365-294X.2005.02553.x 15969739

[B35] Pritchard JK Stephens M Donnelly P Inference of population structure using multilocus genotype data Genetics 2000 155 2 945 959 10835412 10.1093/genetics/155.2.945 PMC1461096

[B36] Excoffier L Laval G Schneider S Arlequin (version 3.0): an integrated software package for population genetics data analysis Evol Bioinform Online 2005 1 47 50 19325852 PMC2658868

[B37] Slatkin M A measure of population subdivision based on microsatellite allele frequencies Genetics 1995 139 1 457 462 7705646 10.1093/genetics/139.1.457 PMC1206343

[B38] Gaggiotti OE Lange O Rassmann K Gliddon C A comparison of two indirect methods for estimating average levels of gene flow using microsatellite data Mol Ecol 1999 8 9 1513 1520 10.1046/j.1365-294x.1999.00730.x 10564457

[B39] Mantel N The detection of disease clustering and a generalized regression approach Cancer Res 1967 27 2 209 220 6018555

[B40] Ovenden JR Peel D Street R Courtney AJ Hoyle SD Peel SL Podlich H The genetic effective and adult census size of an Australian population of tiger prawns (Penaeus esculentus) Mol Ecol 2007 16 1 127 138 17181726 10.1111/j.1365-294X.2006.03132.x

[B41] Wang ZJ Xiong GH Guan LR The achievement of Leishmaniasis control in China Chin J Epidem 2000 21 1 51 54

[B42] Guan LR Wang J Liu PZ Chang ZG The Bionomics of *Phleboomus chinensis* in the nountainous regions of southern Kansu and the Loess Plateau of northern Shensi Acta Entomol Sin 1980 23 1 25 31

[B43] Della Torre A Fanello C Akogbeto M Dossou-yovo J Favia G Petrarca V Coluzzi M Molecular evidence of incipient speciation within *Anopheles gambiae s. s.* in West Africa Insect Mol Biol 2001 10 1 9 18 10.1046/j.1365-2583.2001.00235.x 11240632

[B44] Gentile G Slotman M Ketmaier V Powell JR Caccone A Attempts to molecularly distinguish cryptic taxa in Anopheles gambiae s. s Insect Mol Biol 2001 10 1 25 32 10.1046/j.1365-2583.2001.00237.x 11240634

[B45] Lawniczak MK Emrich SJ Holloway AK Regier AP Olson M White B Redmond S Fulton L Appelbaum E Godfrey J Widespread divergence between incipient *Anopheles gambiae* species revealed by whole genome sequences Science 2010 330 6003 512 514 10.1126/science.1195755 20966253 PMC3674514

[B46] Ma Y Bu L Hua X [20-year search on molecular markers of Leishmania isolates from different Kala-azar foci in China to confirm whether genetic fingerprints of Kala-azar pathogens correlate with disease types] J Biomed Engineer 2011 28 5 997 1000 22097271

